# Applications of Mass Spectrometry in the Onset of Amyloid Fibril Formation: Focus on the Analysis of Early-Stage Oligomers

**DOI:** 10.3389/fchem.2020.00324

**Published:** 2020-05-05

**Authors:** Jiaojiao Hu, Qiuling Zheng

**Affiliations:** Key Laboratory of Drug Metabolism and Pharmacokinetics, State Key Laboratory of Natural Medicines, Department of Pharmaceutical Analysis, School of Pharmacy, China Pharmaceutical University, Nanjing, China

**Keywords:** mass spectrometry, amyloid fibril, aggregation mechanism, early-stage oligomers, conformational analysis

## Abstract

Amyloid fibril formation is a hallmark of diverse neurodegenerative and metabolic diseases, such as Alzheimer's disease (AD), Parkinson's disease (PD), and type 2 diabetes mellitus (T2DM). Conventional diagnosis is based on the appearance of fibrils or plaques, while neglects the role of early-stage oligomers in the disease progression. Recent studies have uncovered that it is the early-stage oligomer, rather than the mature fibril, that greatly contributes cytotoxicity. The formation of oligomers involves complicate structural conversions and it is essential to investigate their conformational changes for a better understanding of aggregation mechanism. The coexistence of soluble early-stage oligomers, intermediates, and pre-fibril species makes it difficult to be differentiate by morphological methods, and only average structural information is provided as they lack the ability of separation. Therefore, mass spectrometry (MS) becomes an alternative technique that presents new and complementary insights into the onset of amyloid fibrils. This review highlights the hotspots and important achievements by MS in the field of amyloid formation mechanism, including the direct detection and differentiation of soluble oligomers (native MS), unambiguous identification of interacted sites involved in the onset of aggregation [hydrogen/deuterium exchange (HDX) and chemical cross-linking (CX)], and conformational switch that leads to fibrilization [collision cross section (CCS) regularity by ion mobility (IM)].

## Introduction

Amyloid fibril formation is a hallmark of numerous neurodegenerative and metabolic diseases, such as Alzheimer's disease (AD), Parkinson's disease (PD), and type 2 diabetes mellitus (T2DM) (Chiti and Dobson, 2017). *In vitro* studies suggest that most amyloid fibril formation follows nucleated polymerization mechanism, involving lag phase and growth phase before mature fibril formed. It has long been recognized that mature fibril is the leading cause of related diseases and techniques are mainly applied for diagnosis of its appearance.

With the development of electron microscopy, such as transmission electron microscope (TEM) and atomic force microscope (AFM), amyloid fibrils ranging from 6 to 130 Å in width and 1,000 to 16,000 Å in length could be detected (Sipe and Cohen, 2000). Dyeing methods, including thioflavin T (ThT) and Congo red, enable the measurement of β-sheet in solution and are applied for kinetic monitoring of aggregation (Munishkina and Fink, 2007). Besides, solid-state nuclear magnetic resonance (ss-NMR) offers molecular level information of amyloid fibrils with its superiority of interaction distance determination, placing constraints on the backbone and side-chain torsion angles, as well as tertiary and quaternary contact identification (Tycko, 2011). X-ray diffraction (XRD) is another high-resolution technique which allows the investigation of molecular structure at the atomic level by obtaining folding information through reflection or transmission diffraction (Nelson et al., 2005).

Recent studies uncover that early-stage oligomer, rather than mature fibril, is the one that greatly contributes to cytotoxicity (Stefani, 2012). However, the physicochemical properties of early-stage oligomer are significantly different from that of fibrils, and no fibril formation makes it difficult to be monitored by the above-mentioned morphological techniques. Though dyeing methods support the solution-based detection, early-stage oligomers with no sufficient β-sheet still could not be sensed. What's more, the coexistence of oligomers, intermediates, and pre-fibril species during early stage yields a heterogeneous solution state and increases detection complexity. Therefore, sensitive and high spatial resolution methods are urgently required.

Mass spectrometry (MS) is a powerful analytical technique that attracts increasing attention in bioanalytical fields. Molecules are converted to gaseous ions in the ion source and separated according to their mass-to-charge ratios (*m/z*) in the mass analyzer. Besides measuring the *m/z*, structural information is provided by tandem MS through dissociation (Domon and Aebersold, 2006). Therefore, MS enables the identification of oligomerization number (the number of monomers that consist of the oligomer, denoted as *n*) according to molecular weight measurement. Different conformations of the same oligomer are discovered upon gas-phase separation, which are derived from formation pathways. On the basis, MS has emerged as a highly sensitive technique for amyloid aggregation studies and presents new and complementary insights into the onset of amyloid fibrils, including oligomer characterization, aggregation mechanism investigation, and inhibitor screening. This review surveys the combination of MS with selected analytical techniques and their advanced achievements in aggregation studies (selected studies are listed in Table 1).

**Table 1:** Representative studies done by mass spectrometry (MS)-based techniques for early-stage oligomer analysis.

**Protein/peptide**	**MS-based** **techniques**	**Achievements**	**References**
Aβ_1−42_	Native top-down	Interaction between Aβ_1−42_ and metal ion	Lermyte et al., 2019
NNQQNY VEALYL SSTNVG	ESI-IM-MS	Mechanism of peptide assembly	Bleiholder et al., 2011
hIAPP Aβ_1−40_	ESI-IM-MS	Mechanisms and binding patterns of small molecule inhibitors	Young et al., 2015; Hoffmann et al., 2017
PrP	HDX-MS	Amyloid fibril formation mechanism	Singh and Udgaonkar, 2013
β2m	HDX-MS	Mechanism of Cu(II) induced amyloid formation	Borotto et al., 2017
Aβ_1−42_	FPOP-MS	Conformational change of the aggregation process	Li et al., 2016
Sup 35	CX-MS	Mechanism of different folding patterns	Wong and King, 2015
α-crystallin	CX-MS	Mechanism of αA66-80 peptide induced aggregation	Kannan et al., 2013

*A_1−42_: Amyloid β_1−42_; hIAPP: human islet amyloid polypeptide; Aβ_1−40_: Amyloid β_1−40_; PrP: prion protein; β2m: β2-microglobulin; ESI: electrospray ionization; IM: ion mobility; HDX: hydrogen/deuterium exchange; FPOP: fast photochemical oxidation of proteins; CX: Chemical cross-linking*.

## Mass Spectrometry

### Native MS

Electrospray ionization (ESI) is one of the soft ionization methods, which has been widely used for protein ionization. During ESI, protein molecules are transferred from solution to the gas phase with the assistance of high voltage (2.5–3.0 kV) and nebulization gas (e.g., N_2_). Denaturant, such as organic solvent, is usually added to enhance the MS signal and achieve high detection sensitivity. However, it is not compatible with holding non-covalent bindings within oligomers, and it will cause dissociation during ionization. Therefore, native MS is an alternative ionization method that enables the direct detection of oligomers (Mitra, 2019). The mild ionization process and the usage of volatile ammonium-based buffer (e.g., ammonium acetate) instead of denaturant not only maintain non-covalent bindings within oligomers but also hold interactions between ligands and oligomers (Bereszczak et al., 2012). For instance, Collingwood and coworkers successfully investigated complexes formed between amyloid protein and nine metal ions by native MS. On the basis, intact oligomer or complex ions are subjected to tandem MS and resulted fragments reveal the information of binding/interacting sites (top-down strategy) (Tipton et al., 2011; Gregorich and Ge, 2014). Two histidine residues near the N-terminus (His 6, His 13) of Aβ_1−42_ were identified as major binding regions by observation of corresponding fragment ions upon electron capture dissociation (ECD), infrared multiphoton dissociation (IRMPD), and collision-induced dissociation (CID) (Lermyte et al., 2019). Native MS serves as a promising method for the detection of intact assemblies. In most cases, the corresponding oligomeric states can be established according to their *m/z* and isotopic distributions. However, there are still cases that species are overlapped and could not be well differentiated: (1) the overlapped species have significant intensity difference; (2) the overlapped species have high charge states so that the isotopic distributions could not be well resolved; or (3) a single oligomer contains different conformations. Therefore, further separation is required.

### Ion Mobility MS (IM-MS)

**Figure 1:**
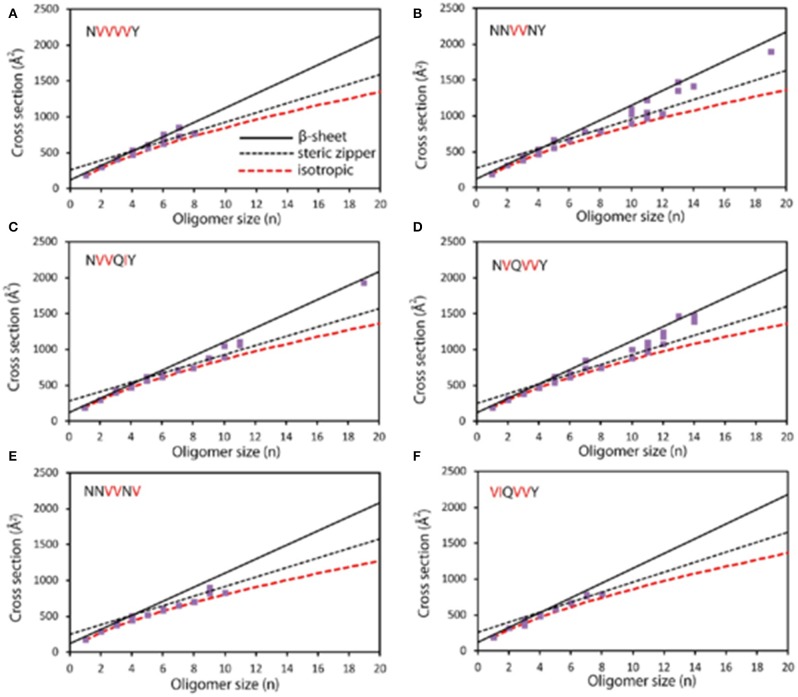
Experimental cross section (Å2) as a function of oligomer size (*n*). **(A)** NVVVVY shows an early transition at *n* = 4; β-sheet structures are observed from *n* = 4 to 7. Structures with steric zipper cross sections are detected at *n* = 6, 7, and 8. **(B)** NNVVNY shows transitions to steric zipper and β-sheet; isotropic cross sections are observed up to *n* = 12 and primarily β-sheet structures for *n* > 12. **(C)** NVVQIY shows a transition to β-sheet at *n* = 10, and β-sheet structures are also at *n* = 19; steric zipper formation also likely appears between *n* = 4 and 11. **(D)** NVQVVY shows a transition from *n* = 7 to 10, with β-sheet structures being observed up to *n* = 14. **(E)** NNVVNV shows isotropic cross section up to *n* = 10 with no β-sheet oligomers and only one steric zipper at *n* = 9. **(F)** VIQVVY shows isotropic cross sections up to *n* = 8 and no larger oligomers. Modified with permission from Do et al. (2013), Copyright 2013 American Chemical Society.

As there are still cases that oligomers overlapped at the same *m/z* or a single oligomer has different conformations, multidimensional separation is essential for further exploration. IM is such a designed technique that enables gas-phase separation of ions based on their mobility in a buffer gas. Gas-phase ions generated from the ion source are subsequently pushed into an IM cell which is filled with a buffer gas (e.g., He). The gas-phase separation depends on their charges, sizes, and shapes, and leads to drift time difference (Kanu et al., 2008). The obtained drift time is then converted to collision cross section (CCS) value to deduce stoichiometry information. Therefore, oligomer ions sharing with the same *m/z*, but with different charges or conformations, could be separated by IM. For instance, MS detection of human islet amyloid polypeptide (hIAPP) showed a doubly charged monomer (denoted a monomer^2+^) predominantly as well as minor dimer^3+^ and trimer^5+^, which were identified according to their *m/z* and isotope distributions. Upon IMS separation, co-populated species sharing the same *m/z* were differentiated according to their CCS values, such as dimer^3+^ overlapped with tetramer^6+^. Moreover, two CCS values of monomer differed by 15% were resolved, referring to compact and expanded conformers (Young et al., 2014). CCS is applied for aggregation mechanism studies by coupling with morphological techniques. The observation of the β-sheet transformation of an aggregating peptide was first achieved by combining with AFM. In this work, based on the CCS values of peptides NNQQNY, VEALYL, SSTNVG, and YGGFL, two assembly pathways of oligomers, including isotopic growth and fibril assembly, were revealed: $\sigma =\sigma_{mon}n^{\frac{2}{3}}$(isotopic growth, σ: the CCS values of oligomer, σ_*mon*_: the CCS value of monomer, and n: the oligomeric state); σ = α*n*+κ (fibril assembly, α: describes the fibril shell area per monomer unite, and κ: means the fibril base given by the fibril diameter) (Bleiholder et al., 2011). Bowers and coworkers also correlated the conformational difference of early-stage oligomer with the structure and morphology of mature fibril by taking six mutants of sup35 (Figure 1) and five mutants of [Leu-5]-Enkephalin as examples (Do et al., 2013, 2014). IM separation connected with infrared spectroscopy (IR) analysis by Pagel and coworkers offered secondary structural analysis of gas-phase ions. This combination overcame the defect that only average structural information could be provided by IR, while no secondary structural information provided by MS. Oligomer ions generated from peptide VEALYL and its variants were first separated by IM and subjected to secondary structural analysis, upon which a positive correlation was revealed between CCS that deviates from isotopic growth and the presence of β-sheets (Seo et al., 2017). The combination of IM-MS and IR also revealed that the hexapeptide NFGAIL followed a nucleation-dependent aggregation mechanism at neural pH (Hoffmann et al., 2018). Besides the investigation of amyloid aggregation, Ashcroft and coworkers performed a high throughput screening of small molecule inhibitors by using IM-MS. Various modes of inhibition were identified, including interaction with different species (monomer or oligomer); through different binding patterns (specific, nonspecific, or colloidal), or different effects on aggregation (monomer binding, binding with different conformations of monomer, or disassembly of oligomers). A novel inhibitor (a nonobvious structural mimetic of chloronaphthoquinine–tryptophan) of hIAPP aggregation was discovered accordingly by screening a library of small molecules (Young et al., 2015; Hoffmann et al., 2017). Therefore, IM-MS serves as a promising method in the analysis of amyloid proteins by revealing conformational information of monomers, differentiating oligomeric states of copopulated species, as well as mechanism study of inhibitor binding (Woods et al., 2013).

### Hydrogen/Deuterium Exchange MS (HDX-MS)

Upon IM, co-populated species could be separated in the gas phase, especially the differentiation of conformational intermediates during amyloid formation. However, it lacks the ability to investigate which region is involved in fibril formation. Therefore, HDX is employed regarding its capability of monitoring protein folding/unfolding dynamics and locating sites or regions that are involved in conformational changes. For HDX, proteins are incubated with D_2_O and amide hydrogens of protein backbone would be replaced by the deuterium in solution. The HDX rate greatly depends on the compactness of the protein as solvent-accessible portions exchange rapidly compared with portions that are buried in the center of the structure (Konermann et al., 2011). For amyloid protein analysis, the formed fibrils were incubated with a deuterated buffer to initiate the exchange and subsequently dissociated to monomer prior to proteolysis and MS analysis (Kheterpal and Wetzel, 2006). Upon HDX, Udgaonkar and coworkers successfully demonstrated the conformation conversion reaction from the cellular prion protein (PrP^c^) to the aggregated form (PrP^sc^). Their work suggested that PrP^sc^ underwent two-step conversions before fibril formation, including the formation of the β-sheet core in region 159–225, and the structure loss in the α1 region for further monomer addition to pre-fibril. The attachment of monomer to fibril induced two-step conformational changes, including helix 1 unfolding only after helices 2 and 3 transformed into β-sheet (Singh and Udgaonkar, 2013). With the combination of covalent labeling-MS using dimethyl(2-hydroxy-5-nitrobenzyl)sulfonium bromide, Vachet and coworkers discovered that only Cu(II) binding induced the *cis-trans* isomerization of the His31-Pro32 amide bond, and increased dynamics in the A, B, D, and E β-strands of β-2-microglobulin, which contributed to the formation of an amyloid-competent dimer interface (Borotto et al., 2017). However, the composition of aggregation converts between monomers and oligomers dynamically, resulting in the coexistence of various intermediates that cannot be captured and resolved by conventional HDX. Thus, kinetic pulse labeling HDX strategy was introduced owing to the capability of the characterization of short-lived aggregates (Carulla et al., 2010). Upon this method, temperature, agitation, and metal ion effects on the Aβ aggregation process were investigated by Gross and coworkers. Their work showed that the center region of Aβ was first to aggregate, and soluble intermediates of lag phase were affected by Cu (II) and temperature (Zhang et al., 2013). These unique advantages make HDX-MS an indispensable weapon on amyloid protein studies, and there are a variety of works done by HDX-MS including coupled with top-down strategy (Pan et al., 2012), monitoring the oligomeric stability of insulin analogs (Nakazawa et al., 2012), aggregation kinetic study of α-synuclein (Illes-Toth et al., 2018), side-chain level oligomer-specific interaction of Aβ (Przygonska et al., 2018), oligomer structure of α-synuclein (Paslawski et al., 2014), and conformational dynamics of β2m (Hodkinson et al., 2012), α-synuclein (Stephens et al., 2018), and tau (Huang et al., 2018). Similarly, there is another chemical labeled method named as fast photochemical oxidation of proteins (FPOP), and it was based on the *in-situ* modification by hydroxyl radicals generated by the pulsed KrF laser (248 nm). With the advantage of fast sampling timescale (~μs) and native or near-native state detection of proteins, FPOP combined with MS can offer high special resolution structural information of proteins or protein complexes (Li et al., 2018). For example, Gross and coworkers applied FPOP-MS to monitor the conformational change and solvent accessibility at various stages in the Aβ_1−42_ aggregation process, and their work showed that the middle domain of Aβ_1−42_ played a major role in aggregation, whereas the N-terminus remained most of its solvent-accessibility during aggregation, and the hydrophobic C-terminus was involved to an intermediate extent (Li et al., 2016).

### Chemical Cross-Linking MS (CX-MS)

HDX-MS is able to define residue or region involved in conformational changes during the aggregation process, yet, tedious sample preparation and back-exchange issue somehow limit its applications. CX appears as an alternative method to monitor protein 3D structure and protein-protein interactions. CX involves bifunctional groups containing a cross-linker that has reactivity toward specific amino acids. The two functional groups are linked by a spacer with defined length so that the side chain of amino acids within this distance could be permanently linked and detected by MS after enzymatic digestion (Sinz, 2018). As CX-MS is compatible with physiological pH, it has been widely used for structural elucidation of aggregates, including the structure of distinct tau monomers (inert and seed-competent) (Mirbaha et al., 2018), β2-microglobulin (Hall et al., 2016), tau (Mirbaha et al., 2015), and prion protein oligomers (Onisko et al., 2005). King and coworkers have applied a photo-reactive cross-linker (p-benzoyl-L-phenylalanine) to probe amino acid proximities of Sup35 prion strains using two mutants. The detection of different cross-link products between two mutants, including intra- and inter-peptide cross-link products between amino acid residues 3/28 and 32/55, clearly indicated different folding patterns (Wong and King, 2015). Additionally, by applying the CX-MS, the interactions between hetero-protein and their effects on protein aggregation can also be obtained. For instance, homobifunctional cross-linker N-hydroxy succinimide esters BS2G and its isotope labeled form were used by Sharma and coworkers to find the specific low molecular weight peptide which induced α-crystallin fibrilization. Results showed that αA66-80 peptide bound to multiple sites in α-crystallin and was close enough to be fixed by BS2G, including the chaperone site, C-terminal extension, and subunit interaction sites. The identification of interactions between αA66-80 peptide and α-crystallin gave a better understanding of protein aggregation mechanism in cataract formation, which would have an impact on therapeutic strategies (Kannan et al., 2013). Another work was done to monitor interactions between β-amyloid with transthyretin monomers and tetramers, which was later confirmed to be biologically relevant. Upon the analysis of cross-linked Aβ and TTR, the modified Lys-15, Lys-76, and Lys-9 of TTR suggested that A strand and EF helix were the major binding sites involving in Aβ and TTR association, which was further confirmed to be significant for biological study of the two proteins (Du and Murphy, 2010).

## Complementary Approaches

There are also complementary approaches that are necessary to study the early stage of amyloid progression. Matrix-assisted laser desorption ionization-MS (MALDI-MS) enables direct detection of oligomers (Severinovskaya et al., 2014). According to the oligomer signals obtained from wild-type Aβ_1−40_ and random sequence Aβ_1−40_ variant, Yeung and coworkers were able to differentiate the peaks of Aβ oligomers generated by MALDI-MS, which came from Aβ complexes assembled in solution rather than random gas-phase aggregation during ionization (Wang et al., 2018). Additionally, MALDI-MS was applied to the inhibition studies. For example, with the detection and characterization of the early stage and nucleating species of Aβ (25–35) aggregation process by MALDI-MS, the myricetin was confirmed to inhibit the aggregation of Aβ (25–35) by delaying the transition from short oligomers to more organized soluble intermediates (Fiori et al., 2013). ^19^F NMR is one of the alternative methods for oligomeric species detection as the chemical shift of the ^19^F nucleus is sensitive to small changes in chemical environment and its detection would not be disturbed by background signals. Taking this advantage, Marsh et al. successfully captured multiple types of oligomers coexisted during the lag phase of aggregation by a ^19^F-labeled Aβ_1−40_. The combination with circular dichroism (CD) and AFM helped obtain more information about secondary structure and confirmed that it was a heterogeneous mixture of oligomers that existed during the lag phase (Suzuki et al., 2013). Additionally, Raman spectroscopy is another method that can provide the secondary structural information of oligomers, which is more sensitive to α-helical signal. Raman microscopy can be applied to the detection of the whole aggregation process starting with soluble species to insoluble mature fibril. Moreover, the combination of Raman microscopy with AFM further explores the changes in secondary structure and correlates it to morphology changes of aggregates. Anderson and coworkers investigated the aggregation process of α-synuclein by the Raman microscopy and AFM, and their work showed that in the aggregation progress, the content of β-sheet increases, while α-helix and disordered secondary structure decreases. The unchanged vibrational spectra during the fibrillization process suggested that a cooperative conformational change would probably contribute to the kinetic control of fibrillization (Apetri et al., 2006). What's more, various fluorescence probes are developed for the identification of prefibril species, but most of them are specially designed for certain amyloid proteins, such as the N-aryl amino naphthalene sulfonate and its analog are designed for the detection of α-synuclein aggregates (Celej et al., 2008). Recently, Carver and coworkers reported a generally applicated fluorescent probe bis (triphenylphosphonium) tetraphenylethene (TPE-TPP), which would emit fluorescence upon the interaction with hydrophobic residues of aggregates. Thus, TEP-TPP dye is more sensitive than ThT dye in the detection of early-stage oligomers as they contain a significant number of exposed hydrophobic residues while lacking sufficient β-sheets (Kumar et al., 2017).

## Conclusion and Future Potentials of MS in Amyloid Research

This review describes the analysis and mechanism studies of amyloid formation by MS coupled with other analytical methodologies, which is complementary to that of biological and morphological methods. Conventional methods offer average structural information by regarding them as an entirety. Early-stage aggregation is actually a mixture of oligomers, which could be clearly differentiated by MS according to their molecular weights. In addition, oligomer ions are further separated in the gas phase by IM so that various conformations are discovered. MS coupling with HDX and CX also enables insight into conformational changes and unambiguous identification of interacted sites involved in the onset of aggregation. However, limitations still exist: (i) current studies are done *in vitro* and results obtained may be different between simulated and real biological systems; (ii) how the mechanism obtained from the simulated system could be verified in real biological systems; and (iii) there are more factors that affect aggregation process during *in vivo* studies which increases the detection complexity.

## Author Contributions

JH did literature research and created the draft. QZ reviewed and revised the manuscript. All authors read and approved the manuscript.

## Conflict of Interest

The authors declare that the research was conducted in the absence of any commercial or financial relationships that could be construed as a potential conflict of interest.
